# The best diagnostic approach for systemic neonatal infections

**DOI:** 10.1186/1824-7288-41-S1-A28

**Published:** 2015-09-24

**Authors:** Roberto Pedicino, Carmela Paciullo, Manuela Bedetta

**Affiliations:** 1 Department of Neonatology, Intensive Neonatal Therapy and Neonatal Pathology, University “La Sapienza di Roma-Policlinico Umberto I”, Roma, 00100, Italy; 2 Department of Intensive Neonatal Therapy, Policlinico Casilino, Roma 00100, Italy

## Introduction

The difficulty to set up a diagnostic model to improve actual medical care results [1], depends on the varieties of clinical presentations for serious infections in the newborn and on his biophysical features.

Additionally, many anamnestic risk factors [2] (chorioamnionitis, positive vagino-rectal colture swabs for GBS) or care risk factors (invasive procedures) potentially involved in the occurrence of neonatal infections, represent furthermore confounding elements that restrain the possibility of redact shared diagnostic Guide Lines.

## Discussion

Following diagnostic methods are available:

1. Clinical Examination of the patient: it still represents a fundamental diagnostic element; even without other data it often leads to the decision to start antibiotic therapy. Besides the classical clinical sings (fever, respiratory distress, etc.) according to some authors, the ECG monitoring of the heart rate could be very important [3,4];

2. Culture test: it is used to “verify” sepsis but for several reasons (contaminations, inadequate blood samples), it doesn't allow to rely exclusively on cultural results for a correct diagnosis: from 14% to 35% of emoculture [5] are negative even if there is a confirmed sepsis (with post-mortem tests or biopsies);

3. Blood cell count: including differential count, it has a low sensibility to contribute in a decisive way to diagnosis. However if leukocytes are less than 5000/mm^3^ diagnosis of serious infection become very suggestive.

4. Inflammatory Markers: many biomarkers tested in research gave a lot of aspectative not confirmed in the clinical practice. Anyway, C-Reactive Protein (to monitoring the effectiveness of therapy) and Procalcitonin (for fast increasing at the onset of sepsis) are the most used [6,7].

5. Molecular Tests: the PCR is an important technology. It can't replace the results of culture test. The main limits are represented by the cost and the impossibility to produce a susceptibility testing[5].

6. Genetics Tests: testing the genetic heritage [8-10] and the gene-expressions of patients (molecular and protein products) is the most recent field of research used to identify patients with a higher risk to develop infections. However, the limits and their true possibilities for clinical application are still unclear.

## Conclusions

In the last 20 years, few results has been reached in reducing mortality due to neonatal infections despite the increased amount for general care and the effort expended on research. Actually, the best diagnostic approach seems still to rely on clinical examination, culture and hematological parameters (leukocytes count, neutrophils count, C-Reactive Protein and Procalcitonin). Promising prospects may be offered in the future from human genetic studies, for all the biological results (proteomics, metabolomics and transcriptomics) that they promise to reveal.
